# Viral Hepatitis in Cambodia: Past, Present, and Future

**DOI:** 10.5005/jp-journals-10018-1165

**Published:** 2016-07-09

**Authors:** Bun Sreng, Chhith Sophal, Sok Srun, Chham Samnang, Eng Huot

**Affiliations:** 1 Department of Communicable Disease Control, Ministry of Health, Phnom Penh, Cambodia; 2 Department of Mental Health, Phnom Penh, Cambodia; 3 Department of Hospital Services, Phnom Penh, Cambodia; 4 World Health Organization, Phnom Penh, Cambodia; 5 Ministry of Health, Phnom Penh, Cambodia

**Keywords:** Hep B vaccine, Infection prevention and control, People Who Inject Drugs (PWIDs), Viral hepatitis.

## Abstract

The prevalence of viral hepatitis represents Cambodia as 1 of the highest endemic countries in the world. But the prevalence of viral hepatitis B among 5-year-olds is on a decreasing trend due to the effect of nationwide vaccination against hepatitis B. There are key interventions to address viral hepatitis by the Cambodian Ministry of Health. There are so far no comprehensive national guidelines for the prevention, care, and treatment of viral hepatitis.

In 2014, the coverage of hepatitis B vaccine at birth and HepB3 vaccine was very high, at 87 and 97% respectively. Other interventions include the screening of viral hepatitis B and C among blood donors; the enhancement of infection prevention and control at health facilities; the program for People Who Use Drugs (PWUDs) and People Who Inject Drugs (PWIDs); control and prevention of HIV/AIDS and sexually transmitted diseases (STDs); and improved sanitation, clean water, and food safety.

The diagnosis of viral hepatitis B and C could be made only at the national and provincial referral hospitals and there are no specific antiviral therapies for people who are infected with hepatitis B and C.

**How to cite this article:**

Sreng B, Sophal C, Srun S, Samnang C, Huot E. Viral Hepatitis in Cambodia: Past, Present, and Future. Euroasian J Hepato-Gastroenterol 2016;6(1):45-48.

## INRODUCTION

Cambodia has struggled with civil war for about 3 decades starting from 1970s through 1990s that left hundreds of thousands people dead. The health infrastructures were severely damaged, including the human resources for health. The first general election was held in 1993 with the support of the United Nations (UN).

Since then, the health system has developed remarkably. So far, Cambodia has 97 hospitals across the country located at national and subnational levels (provincial and district referral hospitals). The number of the health centers and health posts has increased to 1,103 and 106 respectively. The referral hospitals apply Complementary Packages of Activities (CPA) and the health centers apply the Minimum Package of Activities (MPA).

## EPIDEMIOLOGY

The burden of viral hepatitis in the general population has not been well understood as the national prevalence was never carried out, except in the 5-year-old population. Various surveys were done with different sample sizes, specific populations, methods, and locations, at various time periods.

### Viral Hepatitis A

The prevalence of viral hepatitis A increases with age and reaches 100% when the age of the population is 16 years and above.^1-3^ But there have been no documented outbreaks of viral hepatitis A in the country.

### Viral Hepatitis B

According to the WHO’s estimate, the prevalence of viral hepatitis B in Cambodia ranges between 5 and 10%.^4^ Different surveys in the country showed that its prevalence varied between 4.6 and 10.8%.^5,6^ Its prevalence has a decreased trend in the 5-year-old population. A survey in 2006 showed the prevalence of hepatitis B among 5-year children was 3.5% (2.4–4.8%) in 2006 and was between 0.33 and 3.45% in 2012.^7,8^ The prevalence was almost 50% among the populations with liver diseases including high transaminase levels, liver cirrhosis, and liver cancers.^9,10^

### Viral Hepatitis C

Different surveys carried out in Cambodia found the prevalence of viral hepatitis C ranged from 0.7 to 14.7%. It was higher (21%) in patients with hepatocellular carcinoma (HCC) and in patients with high aspartate aminotransferase (AST) and alanine aminotransferase (ALT) (39%).^5,9,10^

### Viral Hepatitis D

Data on viral hepatitis D were not available.

### Viral Hepatitis E

It was found that the prevalence of viral hepatitis E virus (HEV) was 5.5% in patients with increased AST and ALT.^9^

## PREVENTION

### Vaccination

The National Immunization Programme (NIP) has incorporated hepatitis B vaccine into the vaccination schedule starting in 2 provinces, namely Kampong CHHAM and Kampong Cham, in 2000 and then scaled up countrywide in 2005. The coverage of hepatitis B dose at birth and less than 24 hours post birth increased remarkably from 24% in 2005 to 87% in 2014 and the coverage of DPT-HepB-Hib3 reached 97% in 2014.^11^

This resulted from an increase in deliveries at health facilities (22% in 2005 and 83% in 2014).^12^ Other contributing factors included fixed-site immunization at both public and private health facilities, through outreach activities, and vaccination for high-risk communities (four times vaccination per year).

**Fig. 1:**
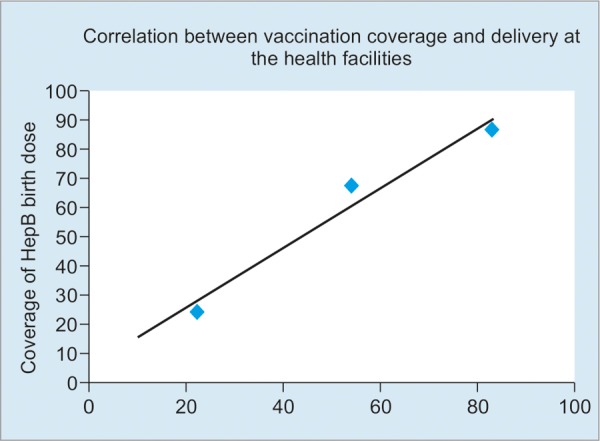
Vaccination coverage *vs* delivery at health facilities

Figure 1 shows the correlation between vaccination coverage and delivery at health facilities in Cambodia.

### Blood Safety

The Ministry of Health has the policy to screen all donated bloods. The National Blood Transfusion Services (NBTS) and blood banks and blood depots have been set up at national and provincial levels. There are around 60,000 blood donors per year.

### Infection Prevention and Control

The national guidelines for infection prevention and control (IPC) were endorsed by the Ministry of Health, which is being coordinated and implemented by health facilities. The Department of Hospital Services provides trainings and monitor and evaluate the IPC practices.

In the late 1990s, comprehensive health education programs aimed at preventing human immunodeficiency virus and acquired immune deficiency syndrome were initiated targeting health professionals and the general public. The Law on the Prevention and Control of HIV/AIDS was enacted by the National Assembly on June 14, 2002.

### Drug Users

The National Authority for Combating Drugs (NACD) was established in 1995 and the national strategic plan was developed. The NACD estimated that there were around 6,000 People Who Inject Drugs (PWIDs) in Cambodia in 2008. According to the Integrated Behavioral Biological Survey (IBBS) conducted in 2012, the number of People Who Use Drugs (PWUDs) was 13,000 (12,000–28,000) and that of PWIDS was 1,300.

The Department of Mental Health and Addiction Services reported that the number of PWIDs and PWUDs who sought treatment at health facilities increased gradually from 150 in 2011 to 282 in 2014 and from 1,669 in 2011 to 2,273 in 2014 respectively.

**Fig. 2:**
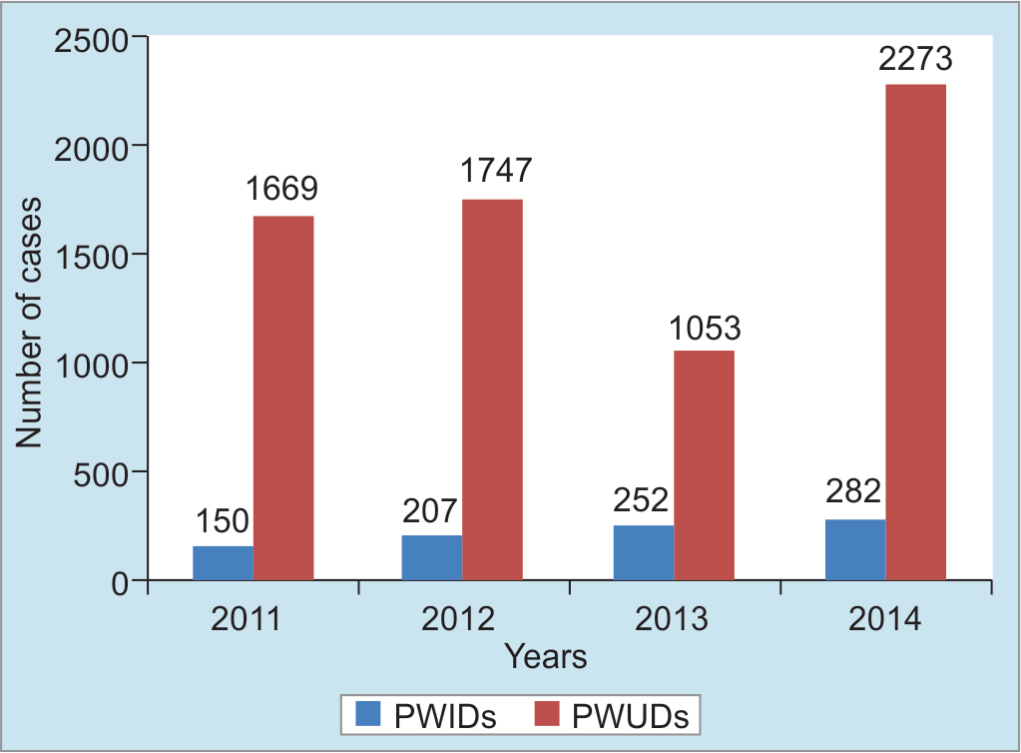
People Who Inject Drugs in Cambodia who sought treatment at public health facilities from 2011 to 2014

Figure 2 shows the number of PWIDs in Cambodia who sought treatment at public health facilities from 2011 to 2014.

### Sexual Transmission

Cambodia has been very successful in the prevention and control of HIV/AIDS and sexually transmitted infections (STIs). The HIV prevalence and incidence decreased remarkably from 1990 to 2010.^13^ This success would have an impact on the reduction of the transmission of viral hepatitis B and C as well.

In the late 1990s, the comprehensive health education programs aimed at preventing HIV and AIDS (health professionals and general public) and the national strategy to eliminate HIV by 2020 were developed.

**Fig. 3:**
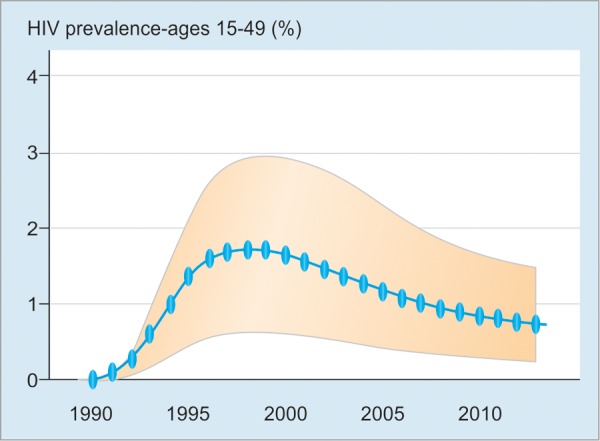
High prevalence of HIV in Cambodia

Figure 3 shows the HIV prevalence in Cambodia for age 15 to 49 years (%).

### Sanitation, Clean Water, and Food Safety

In 2014, the Cambodia Demographic Health Survey showed that almost 50% of the households have improved, non-shared toilet facilities and 85% have a place for hand washing (66% in 2010).^12^

The access to improved water sources increased to 65.2% (Urban = 95%, Rural = 60.1%) during dry season. Fifty-five percent of the households boil water before drinking.^12^

In 2010, the Inter-Ministerial Prakas to enhance food safety was developed and signed by 6 ministers including the Minister of Agriculture, Forestry, and Fisheries; the Minister of Industry, Mining, and Energy; the Minister of Commerce; the Minister of Tourism; the Minister of Health; and the Minister of Economy and Finance with defined roles and responsibilities. A standard operating procedure (SOP) for foodborne diseases entitled “Intersectoral Outbreak Investigation and Response” was developed.

## DIAGNOSIS

So far, the Ministry of Health did not have the national comprehensive guideline specific for the prevention, care, and treatment of persons with chronic hepatitis B and hepatitis C infection. But the surveillance using clinical signs and symptoms was set up for the health centers and the referral hospitals in order to detect and respond to the outbreak of viral hepatitis (Cambodia Early Warning System).

The current Clinical Practice Guidelines for Pediatrics (CPA) does not include viral hepatitis.

Diagnosis methods are available only at national and provincial hospitals. The immunochromatographic tests for the detection of the hepatitis B surface antigen in plasma and serum and of antibody against hepatitis C virus in human serum, plasma, or whole blood have been accomplished. But the health centers that are the frontline health facilities do not have access to the tests.

## TREATMENT

The treatment is basically symptomatic and there are no antiviral therapies for chronic hepatitis B and C due to its unavailability in the Essential Drug List (EDL) of the Ministry of Health. Antiviral therapies for viral hepatitis B and C can be sought by patients at their own cost.

## CONCLUSION

The burden of viral hepatitis in Cambodia was very high. The current fragmented interventions would contribute to reducing the prevalence of viral hepatitis. Comprehensive national guidelines for the prevention, care, and treatment of viral hepatitis are needed.
